# Unpacking the “Black Box”: How an SMS-Based Continuing Medical Education Intervention Improved Medical Knowledge Among HIV Clinicians in Vietnam

**DOI:** 10.9745/GHSP-D-18-00298

**Published:** 2018-12-27

**Authors:** Maia R. Nofal, Nafisa Halim, Bao Ngoc Le, Lora L. Sabin, Anna Larson Williams, Rachael Bonawitz, Ha Viet Nguyen, Tam Thi Thanh Nguyen, Christopher J. Gill

**Affiliations:** a Department of Global Health, Boston University School of Public Health, Boston, MA, USA.; b Consulting, Researching Community Development (CRCD), Hanoi, Vietnam.; c Center for Population Research Information and Databases (CPRID), General Office for Population and Family Planning, Ministry of Health, Hanoi, Vietnam.

## Abstract

**Background:**

A mobile-based continuing medical education (mCME) intervention implemented over 6 months between 2016 and 2017, consisting of daily SMS multiple choice quizzes and access to online daily readings and CME courses, was shown to be effective in increasing medical knowledge among HIV providers in Vietnam. We hypothesized this improvement was a result of “lateral learning,” a process in which the daily SMS quizzes acted as a stimulus for interacting with other study materials.

**Methods:**

We explored how study materials directly provided by the intervention—the daily readings and the online CME courses—and independent study behaviors, such as using medical textbooks and reviewing national guidelines, contributed to medical knowledge as measured by baseline and endline exams. At baseline, there were 53 participants each in the intervention and control groups (N=106). Using linear regression models, we estimated the association between intervention-prompted and independent study behaviors and endline test scores. We also conducted a series of interaction analyses to test the extent to which the effect of daily quiz performance on endline test scores depended on use of the intervention-prompted or independent study materials. Finally, we estimated the proportion of variance in endline test scores explained by each of the intervention-prompted behaviors.

**Results:**

The average medical knowledge test score among all participants was 46% at baseline and 54% at endline. Among the intervention group, 82% of the daily quizzes were answered, although only about half were answered correctly. Responding to the daily quizzes (ß=0.24; *P*=.05), quiz performance (ß=0.42; *P*<.001), and accessing daily readings (ß=0.22; *P*=.06) were statistically significantly associated with higher endline test scores. While accessing the online CME courses and some of the independent study behaviors, such as use of medical textbooks, had positive associations with endline test scores, none reached statistical significance. Quiz performance explained 51% of the variation in endline test scores. Interaction analysis found that quiz performance had a stronger, but not statistically significant, association with endline test scores when both daily readings (ß=0.87; *P*=.08) and online CME courses (ß=0.25; *P*=.09) were accessed more frequently.

**Conclusion:**

In mCME interventions, daily SMS quizzes can effectively act as a stimulus for uptake of study behaviors when paired with access to relevant readings and online courses. While further investigation is needed to more fully understand the role of outside study materials, we believe this model has the potential for further use in Vietnam and other low-resource settings.

## Abstract (precis)

Daily SMS quizzes sent to medical practitioners seem to act as a stimulus for further self-study when paired with access to additional readings and online courses, improving medical knowledge as a result.

## INTRODUCTION

In 2009, the Vietnamese government passed a law requiring medical practitioners to participate in continuing medical education (CME) to maintain their licensure.^1^^,^^2^ Given the costs and complexity of traditional in-person CME workshops, the Vietnamese Ministry of Health (MOH) selected distance learning as the optimal strategy for supporting the quality of its clinical workforce.^3^^–^^6^

Between 2014 and 2017, in partnership with the Vietnamese MOH and Hanoi Medical University, we developed, tested, evaluated, and refined a mobile CME (mCME) intervention using short message service (SMS) to improve the medical knowledge of clinicians in Vietnam.^7^^,^^8^ In the final iteration of the intervention (mCME version 2.0), conducted over a 6-month period between 2016 and 2017, the intervention group received an SMS with a multiple choice question once daily along with a reply congratulating for correct answers or encouraging better luck next time while providing the correct answer. In all cases, the intervention group received a daily hyperlink to technical readings related to the quiz question, typically 13 paragraphs in length, and invitations to participate in online CME courses hosted by Hanoi Medical University. The control group received a weekly SMS without any medical content that simply reminded them that they were a study participant, and did not receive the links to daily readings or invitations to take the CME courses. However, both the intervention and control group were introduced to the online CME courses at baseline, were given access to the courses, and were encouraged to take them on their own schedule. At the end of the experiment, intervention participants significantly outperformed the controls on the endline examination.^9^

We theorized that the intervention group's SMS messages served as stimuli to motivate broader learning, a process that we called “lateral learning,” but that is arguably a pedagogical adaptation of the Health Belief Model.^10^^,^^11^ The Health Belief Model posits that individuals are likely to change behavior if they believe that they are self-efficacious or that they can successfully complete the behavior of interest despite barriers.^12^ Individuals' self-efficacy is a by-product of 4 factors: the individuals' perception that they are susceptible to a condition (perceived susceptibility), which could have severe consequences (perceived severity), and the individuals' belief that the behavior of interest would lead to more benefits (perceived benefits) than costs (perceived barriers).^12^^,^^13^ Also, the model talks about cues to action, which are referred to as those factors that serve to stimulate or prompt behaviors.^14^ Cues to action set in motion the process of behavior change, and without any cues, an individual may delay a behavior change despite perceptions of susceptibility, severity, and benefits outweighing barriers.^14^

In an mCME intervention, we theorized medical quizzes sent via SMS to clinicians served as stimuli to motivate broader learning, a process we called lateral learning.

The goal of this analysis was to better understand how the different components of the mCME version 2.0 intervention, alone or in combination, contributed to medical knowledge gains—in other words, unpacking the “black box” (Figure). Specifically, we addressed the following questions:

1. Did the mCME version 2.0 intervention improve the endline exam scores by encouraging uptake of intervention-prompted study behaviors?
2. Did the mCME version 2.0 intervention improve the endline exam scores by encouraging uptake of independent study behaviors outside of those directly prompted by the intervention?

**FIGURE:**
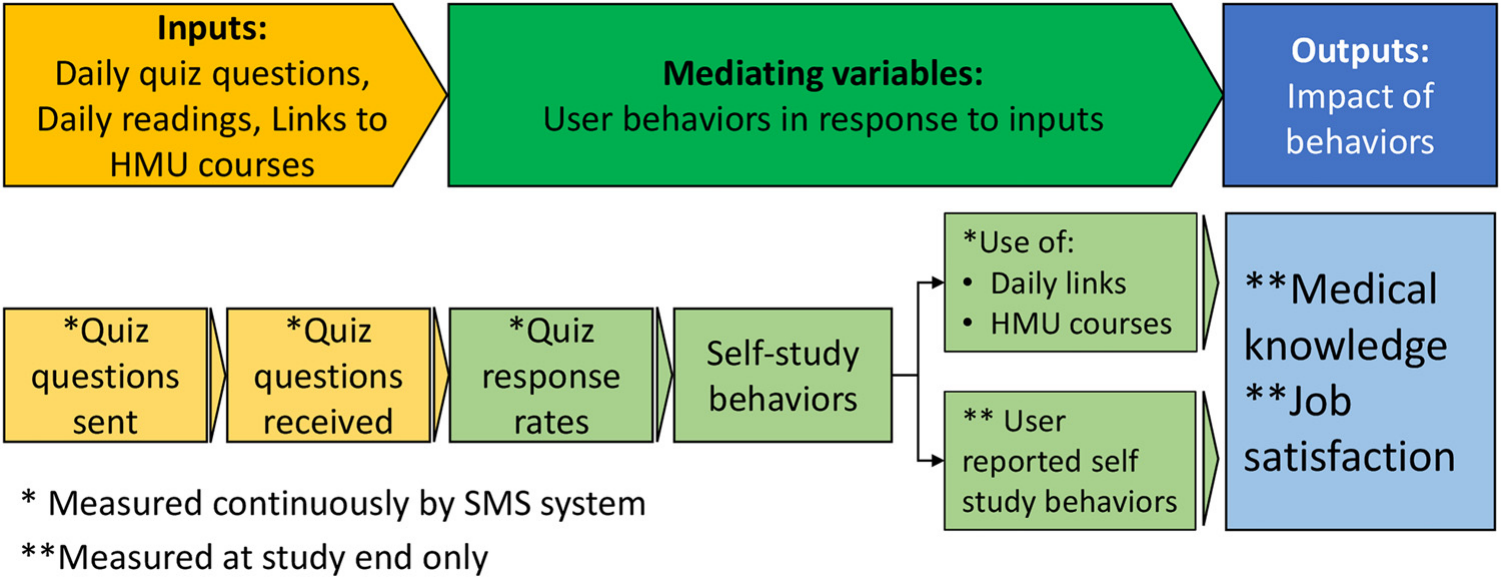
Logic Model of Lateral Learning for the mCME Intervention in Vietnam Abbreviations: HMU, Hanoi Medical University; mCME, mobile continuing medical education; SMS, short message service. The mCME intervention implemented in Vietnam was hypothesized to function as a pedagogical analog to the Health Belief Model. In the intervention, the stimuli were the daily text messages and the desired initial outputs were a change in self-study behaviors, which were also a hypothesized mediator of acquired medical knowledge. As in the Health Belief Model, the potency of the stimuli were contingent on the willingness of a given individual to respond by investing their time and energy to self-study.

## METHODS

### Study Design Overview

Full details of the mCME methodology have been published previously.^8^^,^^9^ The intervention, conducted over a 6-month period between 2016 and 2017, consisted of sets of daily SMS quiz questions, linked readings, and invitations to participate in online CME courses on the same topics, clustered thematically into 15 modules covering a range of clinical topics in clinical HIV/AIDS care. The daily SMS quiz questions were developed in English, which were then translated into Vietnamese for delivery. All other intervention components were developed and delivered in Vietnamese. The control group had access to the online CME courses but did not receive the daily SMS quizzes or other messages encouraging self-study. In total, 106 HIV clinicians participated in the study (n=53 in the intervention group, n=53 in the control group).

### Data, Measures, and Variables

*Outcome variables*: Our outcome variable was HIV knowledge at the endline examination. We measured HIV knowledge through a 100-item standardized, multiple choice test focused on aspects of HIV care covered in the 15 online CME courses.

*Intervention-prompted behaviors:* Data were captured in real time on participants' use of the daily quizzes; their performance on the daily quizzes; access of the daily hyperlinked readings; and access of the online CME courses.

*Independent study behaviors*: Outside of using the study materials directly provided through the intervention, we were interested in how the intervention affected the uptake of additional study materials. We defined the use of these study materials as “independent study behaviors.” These were behaviors that were not directly encouraged by the intervention but may have had a significant impact on the participants' endline score. To assess this, we collected self-study behaviors via a survey administered at the endline evaluation. This survey assessed levels of engagement (4 or more times/week; 1–3 times/week; 1–3 times/month; <1 time/month) and changes (more, less, or the same, compared with before the mCME study) pertinent to 6 types of study habits:

1. Using medical textbooks.
2. Consulting with colleagues.
3. Researching information online using Google or another web browser to refresh clinical knowledge.
4. Using websites developed specifically for medical professionals.
5. Reviewing the official guidelines for HIV practice issued by the Vietnamese MOH.
6. Reviewing scientific research papers from the medical literature.

*Control variables* included baseline exam scores on the HIV knowledge examination and a number of sociodemographic attributes. Similar to the endline exam, baseline exam scores were based on a multiple choice test of medical knowledge. While the baseline and endline exams covered the same 15 thematic areas covered in the online CME courses, the exam questions were not repeated. Further, we adjusted for participants' gender and age collected via self-report at baseline. Gender was measured as a binary variable with female as the reference category, and age was measured as a continuous variable.

### Data Analysis

Attempting to answer our 2 research questions, we estimated the extent to which endline test scores were associated with intervention-prompted and independent study behaviors. All regression models were adjusted for gender, age, years of experience in HIV-care provision, and baseline test scores. The sample size included in the analysis depended on whether the endpoint pertained to the full cohort (intervention and control participants, N=106), or solely the intervention group at baseline (n=53) or the intervention group retained at endline (n=48).

To answer our first question, how the 4 components of the intervention (SMS quizzes, quiz performance, daily readings, and online CME courses), affected gains in medical knowledge, we fit 6 adjusted linear regression models to estimate the association between intervention-prompted behaviors and endline test scores, one model for each of the 4 intervention-prompted behaviors and 2 models for all 4 together. To address our second question, how the intervention affected independent study behaviors in order to affect gains in medical knowledge, we fit 4 adjusted linear regression models, 2 to test the independent study behaviors as effect mediators and 2 to test the differential effects of independent study behaviors by intervention status.

Finally, we conducted 2 additional analyses to further explore our core findings pertinent to intervention-prompted behaviors. First, we tested the extent to which the effect of quiz performance on endline test scores may depend on utilization of daily readings, online CME courses, or self-study resources. For that, we conducted a series of interaction analyses. Second, we estimated the proportion of variance in endline test scores explained by each of the intervention-prompted behaviors. All analyses were conducted in SAS 9.4.

## RESULTS

### Descriptive Results

On average, the mCME study participants (intervention and control group combined) were 41 years of age and had 4 years of experience in HIV care. Most (56%) were women. The mean medical knowledge test score among both groups combined was 46% at baseline and 54% at endline (Table 1). As noted in the report of the primary study findings,^9^ the small sample size precluded precise measurement of impact on medical knowledge.

**TABLE 1.** Sociodemographic Characteristics and Test Scores of Study Participants (N=106).

	Value
**Female, %**	56.0
**Age, years, mean (SD)**	41.2 (9.5)
**Highest clinical degree, No. (%)**	
CBPA	3 (2.8)
CK1	57 (53.8)
CK2	1 (0.9)
MD	45 (42.5)
**Years of experience providing HIV care to patients, mean (SD)**	4.2 (4.9)
**Number of patients typically seen each daily, No. (%)**	
1–2	62 (58.5)
3–4	13 (12.3)
5–7	12 (11.3)
8–11	8 (7.6)
12+	11 (10.4)
**Baseline scores, mean (SD)**	46.4 (11.9)
Intervention group	44.6 (12.4)
Control group	48.2 (11.2)
**Endline scores,**^**a**^ **mean (SD)**	54.2 (12.1)
Intervention group	55.0 (11.7)
Control group	53.4 (12.5)
**% change in scores between baseline and endline,**^**a**^ **mean (SD)**	19.6 (30.9)
Intervention group	25.6 (32.4)
Control group	13.5 (28.2)

Abbreviations: CBPA, community-based physician's assistants; CK1, first-level specialization/specialist; CK2, second-level specialization/specialist, SD, standard deviation.

a Sample size at endline consisted of 95 individuals (n=48 in the intervention group, n=47 in the control group).

Among the intervention participants over the 6-month study period, on average 82% of the daily quizzes delivered were answered, although only about half of them were answered correctly (Table 2). The timing of the SMS had no effect on participation in daily quizzes: participants were as likely to respond to daily quizzes regardless of whether the SMS was sent in the morning or in the afternoon (82% for 9 am vs. 81% for 1 pm).

**TABLE 2.** HIV Clinicians' Participation in Intervention-Prompted and Independent Study Behaviors

	Value
**Intervention-Prompted Study Behaviors Among Intervention Group (n=53)**	
% of SMS quizzes answered, mean (SD)	81.9 (23.1)
% of SMS quizzes correctly answered, mean (SD)	52.0 (20.5)
% of daily hyperlinks accessed, mean (SD)	18.1 (21.8)
Ever accessed online CME courses,^a^ % (SD)	60.4 (49.4)
**Independent Study Behaviors Among Intervention and Control Groups Combined** ^b^	
Used medical textbooks (ref: 1–3 times/month or <1 time/month)	61.7 (48.9)
Consulted with colleagues (ref: 1–3 times/month or <1 time/month)	44.7 (50.0)
Researched online (ref: 1–3 times/month or <1 time/month)	56.8 (49.8)
Researched website for medical professionals (ref: 1–3 times/month or <1 time/month)	55.3 (50.0)
Reviewed HIV guidelines (ref: 1–3 times/month or <1 time/month)	61.1 (49.0)
Reviewed scientific papers (ref: 1–3 times/month or <1 time/month)	42.6 (49.7)
More use of medical textbooks than before (ref: the same or less)	62.8 (48.6)
More consultation with colleagues than before (ref: the same or less)	43.2 (49.8)
More research online than before (ref: the same or less)	60.2 (49.2)
More visits to researched medical websites than before (ref: the same or less)	56.8 (49.8)
More frequent review of HIV guidelines than before (ref: the same or less)	62.4 (48.7)
More frequent reviews of scientific papers than before (ref: the same or less)	50.5 (50.3)
Total number of study resources used 1–3 or 4 or more times/week, mean (SD)	3.6 (2.7)

Abbreviations: CME, continuing medical education; SD, standard deviation; SMS, short message service.

a Among the entire study group (intervention and control group combined), 43.4% (SD=49.8) ever accessed the online CME courses.

b Sample size for the intervention and control group varied slightly for each indicator, from 93 to 95 individuals, depending on how many respondents answered each question.

On average, 82% of the daily quizzes were answered, but only about half were answered correctly.

The daily hyperlinked readings sent with each quiz had lower participation rates than the daily quizzes, only 20% on average. Of the 53 HIV clinicians assigned to the intervention arm, 41 accessed between 1% and 89% of the hyperlinks while 12 never accessed the hyperlinks. The timing of the SMS had an impact on participants' access of the daily hyperlinks: a greater percentage of participants accessed the daily hyperlinks when the SMS was sent in the morning than in the afternoon (23% for 9 am vs. 13% for 1 pm).

Across both the intervention and control groups, 43% of study participants ever accessed the online CME courses throughout the intervention period. Among the intervention group only, 60% of the study participants ever accessed the online CME courses. In addition, the intervention group accessed the CME courses more times (134 times) than the control group (27 times).

Among the 6 independent study behaviors queried on the endline survey, using medical textbooks and reviewing the Vietnamese HIV guidelines were the most frequently used study strategies (Table 2). Consulting with colleagues and reading scientific papers were the least commonly used self-study behaviors. On average, participants from both groups used 3.62 study resources a week. Most participants also said that they used medical textbooks, researched online, visited medical websites, and reviewed the HIV guidelines more than they had before the study.

Notably, clinicians could engage in these behaviors irrespective of study arm and not all clinicians answered all questions. The variables capturing change in self-study behaviors were based on the questions asked in the endline survey on clinicians' self-assessment of their self-study behavior compared with before the intervention.

### Bivariate Results

All intervention-prompted study behaviors were positively correlated with endline test scores (Table 3, Column 1). Baseline test scores had a positive correlation with only quiz participation and performance (Column 2). Further, endline test scores had the strongest correlation (*r*=0.36) with quiz performance (Column 1, Row 4), and quiz performance had a stronger correlation with endline (*r*=0.36) than baseline test scores (*r*=0.11). Further, quiz performance had a strong positive correlation with quiz participation (r=0.83) and a moderately strong positive correlation with access of daily readings (*r*=0.56).

**TABLE 3.** Correlation Coefficients for Intervention-Prompted Study Behaviors and Baseline and Endline Test Scores (n=48)

		(1)		(2)		(3)		(4)		(5)		(6)		(7)		(8)		(9)	
		Endline Test Scores		Baseline Test Scores		Average % of SMS Quizzes Answered		Average % of SMS Quizzes Correctly Answered		Average % of Daily Readings Accessed		Ever Accessed Online CME Courses		Years of Experience in HIV Care Provision		Age		Male	
		*r*	*P*	*r*	*P*	*r*	*P*	*r*	*P*	*r*	*P*	*r*	*P*	*r*	*P*	*r*	*P*	*r*	*P*
(1)	Endline test scores	1.00																	
(2)	Baseline test scores	0.59	<.001	1.00															
(3)	Average % of SMS quizzes answered	0.17	.26	0.04	.81	1.00													
(4)	Average % of SMS quizzes correctly answered	0.36	.01	0.11	.47	0.83	<.001	1.00											
(5)	Average % of daily readings accessed	0.20	.18	−0.004	.98	0.38	.01	0.56	<.001	1.00									
(6)	Ever accessed online CME courses	0.04	.81	−0.08	.57	0.45	.002	0.35	.01	0.30	.04	1.00							
(7)	Years of experience in HIV care provision	0.16	.28	−0.07	.65	−0.16	.28	-0.09	.56	−0.002	.99	−0.19	.20	1.00					
(8)	Age	−0.13	.39	-0.09	.53	0.13	.36	0.26	.08	0.14	.36	0.07	.63	0.33	.02	1.00			
(9)	Male	−0.12	.41	-0.11	.45	0.19	.20	0.16	.27	-0.17	.26	0.06	.66	0.15	.30	0.23	.11	1.00	

Abbreviations: CME, continuing medical education; SMS, short message service.

All intervention-prompted study behaviors were positively correlated with endline test scores.

### Modeling Results

#### Associations Between Intervention-Prompted Behaviors and Endline Test Scores

Table 4 presents the standardized regression coefficients, which captured the relative association with endline test scores of each of 4 intervention-prompted behaviors, alone (Models 1–4) and combined (Models 5–6). In Model 1, increased quiz participation predicted an increase in endline test scores (ß=0.24; *P*=.05). The percentage of SMS quizzes that participants answered correctly was more strongly predictive of endline test scores: Based on Model 2, a 1 standard deviation increase in the percentage of SMS quizzes that participants answered correctly was associated with nearly a half-point increase in endline test scores (ß=0.42; *P*<.001). The percentage of daily readings accessed, as demonstrated by Model 3, had a positive association with endline test scores when considered alone (ß=0.22; *P*=.06). Similarly, whether or not the participants ever accessed the online CME courses had a positive association with endline test scores in Model 4 (ß=0.16), but the association was not statistically significant (*P*=.19). In the combined models, we included quiz participation and performance alternatively, given their strong correlation (r=0.83, Table 3). When mutually adjusted in Models 5–6, only the percentage of SMS quizzes that participants answered correctly was predictive of endline test scores: as in Model 2, a 1 standard deviation increase in the percentage of SMS quizzes that participants answered correctly was associated with nearly a half-point increase in endline test scores (ß=0.43). In terms of model fitness, Model 2 had the best fit, explaining 51% of variation in endline test scores.

**TABLE 4.** Associations Between Intervention-Prompted Study Behaviors and Total Endline Test Scores (n=48)

	Model 1: Predictor, Quiz Participation		Model 2: Predictor, Quiz Performance		Model 3: Predictor, Daily Readings		Model 4: Predictor, Online CME Courses		Model 5: All Predictors Except Quiz Performance^a^		Model 6: All Predictors Except Quiz Participation^a^	
	ß (SE)	*P*	ß (SE)	*P*	ß (SE)	*P*	ß (SE)	*P*	ß (SE)	*P*	ß (SE)	*P*
Average % of SMS quizzes answered	0.24 (0.06)	.05							0.16 (0.07)	.27		
Average % of SMS quizzes correctly answered			0.42 (0.06)	.004							0.43 (0.08)	.005
Average % of daily readings accessed					0.22 (0.06)	.06			0.14 (0.07)	.28	−0.03 (0.07)	.81
Accessed online CME courses (ref: never)							0.16 (2.89)	.19	0.05 (3.16)	.71	0.03 (2.78)	.79
Male (ref: female)	−0.11 (2.81)	.38	−0.12 (2.51)	.28	−0.01 (2.83)	.92	−0.07 (2.84)	.57	−0.06 (2.97)	0.62	−0.13 (2.74)	.28
Age	−0.18 (0.16)	.15	−0.27 (0.15)	.02	−0.19 (0.17)	.14	−0.16 (0.17)	.21	−0.20 (0.17)	0.11	−0.27 (0.16)	.03
Years of HIV care provision	0.31 (0.30)	.02	0.34 (0.27)	.004	0.26 (0.29)	.04	0.29 (0.31)	.03	0.31 (0.31)	.02	0.34 (0.28)	.005
Baseline test score	0.57 (0.11)	<.001	0.53 (0.10)	<.001	0.59 (0.11)	<.001	0.60 (0.12)	<.001	0.58 (0.11)	<.001	0.53 (0.11)	<.001
Adjusted R^2^	0.40		0.51		0.40		0.37		0.39		0.49	

Abbreviations: CME, continuing medical education; SE, standard error; SMS, short message service.

a Model 5 includes Quiz Participation, Daily Readings, Online CME Courses, and controls while Model 6 includes Quiz Performance, Daily Readings, Online CME Courses, and controls. Quiz Participation and Quiz Performance were not included in the same model together due to high collinearity.

#### Associations Between Independent Study Behaviors and Endline Test Scores

Table 5 presents the associations between independent study behaviors and endline test scores among all participants (Models 1–2); intervention participants only (Model 3); and control participants only (Model 4). In Model 2, we tested the extent to which the association between the intervention status and endline test scores (Model 1) was mediated by independent study behaviors. Additionally, we present the associations between independent study behaviors and endline test scores for intervention participants and control participants by way of assessing the extent to which associations varied by intervention status.

**TABLE 5.** Associations Between Independent Study Behaviors and Total Endline Scores

	Model 1 Predictor, Intervention Status (n=95)		Model 2 Independent Study Behaviors as Potential Mediators of Intervention Effect (n=91)		Model 3 Independent Study Behaviors as Predictors of Endline Scores Among Intervention Group (n=47)		Model 4 Independent Study Behaviors as Predictors of Endline Scores Among Control Group (n=44)	
	ß (SE)	*P*	ß (SE)	*P*	ß (SE)	*P*	ß (SE)	*P*
Treatment status (ref: control)	0.11 (2.08)	.22	0.15 (2.09)	.11				
Used medical textbooks (ref: 1–3 times/month or <1 time/month)			−0.11 (3.11)	.41	0.07 (3.95)	.69	−0.35 (5.47)	.16
Consulted with colleagues (ref: 1–3 times/month or <1 time/month)			−0.12 (2.57)	.28	−0.23 (3.18)	.11	0.18 (4.56)	.35
Researched online (ref: 1–3 times/month or <1 time/month)			0.20 (3.32)	.19	0.26 (3.89)	.11	0.12 (6.56)	.69
Researched website for medical professionals (ref: 1–3 times/month or <1 time/month)			−0.04 (3.11)	.80	−0.14 (3.84)	.41	0.40 (6.07)	.14
Reviewed HIV guidelines (ref: 1–3 times/month or <1 time/month)			−0.09 (2.70)	.45	−0.002 (3.81)	.99	−0.15 (4.37)	.45
Reviewed scientific papers (ref: 1–3 times/month or <1 time/month)			0.17 (2.46)	.12	0.21 (3.13)	.15	−0.03 (4.56)	.89
Male (ref: female)	−0.25 (2.12)	.01	−0.28 (2.16)	.005	−0.15 (2.71)	.23	−0.55 (4.67)	.01
Age	−0.02 (0.12)	.86	0.11 (0.13)	.32	−0.07 (0.19)	.66	0.23 (0.19)	.19
Years of HIV care provision	0.15 (0.24)	.12	0.12 (0.23)	.24	0.23 (0.33)	.11	0.04 (0.37)	.83
Baseline test score	0.50 (0.09)	<.001	0.55 (0.09)	<.001	0.62 (0.12)	<.001	0.47 (0.16)	.01
Adjusted R^2^	0.31		0.34		0.42		0.26	

Abbreviation: SE, standard error.

After adjusting for baseline test scores (which were lower among the intervention than control participants) along with additional controls (Model 1), intervention participants achieved higher endline test scores suggesting that the intervention led to improvement in medical knowledge among HIV clinicians (ß=0.11), but the result was not statistically significant (*P*=.22). The relative association of the intervention grew somewhat stronger when independent study behaviors were added to the model (Model 2) (ß=0.15; *P*=.11). Independent study behaviors appeared to act as moderators of the overall effect of the intervention on endline test scores—when considering the strength of association of the intervention, the regression coefficient increased when independent study behaviors were added to the model.^15^

Among the intervention participants (Model 3), ‘use of medical textbooks’ (ß=0.07; *P*=.69), ‘researched online’ (ß=0.26; *P*=.11), and ‘reviewed scientific papers’ (ß=0.21; *P*=.15) had positive but not statistically significant associations with endline test scores. On the other hand, ‘consulted with colleagues’ (ß=−0.23; *P*=.11), ‘researched website for medical professionals’ (ß=−0.14; *P*=.41), and ‘reviewed HIV guidelines’ (ß=−0.002; *P*=.99) had negative correlations, although these correlations also were not statistically significant. Among the control participants (Model 4), ‘consulted with colleagues’ (ß=0.18; *P*=.35), ‘researched online’ (ß=0.12; *P*=.69), and ‘researched websites for medical professionals’ (ß=0.40; *P*=.14) had positive associations with endline test scores, while ‘used medical textbooks’ (ß=−0.35; *P*=.16), ‘reviewed HIV guidelines’ (ß=−0.15; *P*=.45), and ‘reviewed scientific papers’ (ß=−0.03; *P*=.89) had negative associations. None of these associations were statistically significant.

### Additional Analyses

#### Associations Between Endline Test Scores and Interactions Between Quiz Performance and Independent Study Behaviors

Of all potential variables, the percentage of SMS quizzes answered correctly was the best predictor of endline test scores (Table 4). To better understand this association, we tested the extent to which the effects of quiz performance on endline test scores depended on accessing the daily readings, online CME courses, or self-study resources. Therefore, we conducted a series of interaction analyses: we constructed 8 interaction terms (percentage of quizzes answered correctly multiplied with each of 8 variables including daily readings, online CME course usage, and the 6 independent study behaviors) and used each interaction (e.g., percentage of SMS quizzes correctly answered * used medical textbooks) along with original terms (e.g., percentage of SMS quizzes correctly answered; used medical textbooks) as covariates in adjusted regression models.

Quiz performance had a stronger association with endline test scores when both daily readings and online CME courses were accessed more frequently, suggesting that use of multiple elements of the intervention had added benefits (Table 6). HIV clinicians scored higher endline test scores when they more frequently accessed daily readings (ß=0.87; *P*=.08) or visited online CME course websites (ß=0.25; *P*=.09) as well as better performed in daily quizzes. None of the interactions between quiz performance and each of 6 independent study behaviors were statistically significant suggesting that the effect of quiz performance on endline test scores was not conditional on independent study behaviors.

**TABLE 6.** Associations of Interactions Between Quiz Performance and Study Behaviors With Total Endline Scores

	Model 1 (n=48)		Model 2 (n=48)	
	ß (SE)	*P*	ß (SE)	*P*
% of SMS quizzes correctly answered	0.18 (0.08)	.04	0.11 (0.09)	.24
% of daily readings accessed	−0.64 (0.37)	.09		
Accessed online CME courses (ref: never)			−13.81 (8.06)	.09
% of SMS quizzes correctly answered * % of daily readings accessed	0.87 (0.48)	.08		
% of SMS quizzes correctly answered * accessed online CME courses			0.25 (0.15)	.09
Age	−1.88 (2.85)	.51	−0.92 (2.76)	.74
Years of HIV care provision	−0.28 (0.16)	.09	−0.30 (0.16)	.08
Baseline test score	0.49 (0.11)	<.001	0.52 (0.11)	<.001
Intercept	36.67 (9.15)	<.001	37.69 (9.51)	<.001
Adjusted R^2^	0.44		0.43	

Abbreviations: CME, continuing medical education; SE, standard error; SMS, short message service.

Quiz performance had a stronger association with endline test scores when both daily readings and online CME courses were accessed more frequently.

#### Percentage of Total Explained Variances Attributable to Intervention-Prompted or Independent Study Behaviors

Finally, within the intervention group, we considered how much of the total variance in endline test scores was explained by each of the intervention-prompted or independent study behaviors considered (Table 7). We report on intervention-prompted study behaviors only. For this analysis, we fit a model of endline test scores using gender, age, years of experience in HIV care provision, and baseline score as covariates (Model 1), and report the total variance explained by Model 1 (adjusted R^2^=0.36). Next, we fit Model 2, adding to Model 1 the percentage of SMS quizzes correctly answered, and report the total variance explained by Model 2 (adjusted R^2^=0.51). Third, we calculated the proportion of the total variance explained by the percentage of SMS quizzes correctly answered [((Adjusted R^2^ of Model 2 − Adjusted R^2^ of Model 1)/Adjusted R^2^ of Model 1) * 100]. We followed the same procedure for the remaining 8 intervention-prompted or independent study behaviors. The percentage of total variance explained by intervention-prompted study behaviors ranged between 3% and 43%. SMS quiz performance explained the highest variance in endline test scores, further supporting that this aspect of the intervention-prompted study behaviors was the most important to the success of the intervention. Among the remaining intervention-prompted behaviors, the percentage of daily readings accessed explained more variation in endline test scores than gender, age, years of experience in HIV care provision, and baseline test scores alone: 40% as opposed to 36%, and 10% of that variability was attributable to the percentage of daily readings accessed. Finally, accessing the online CME courses explained 1% more of the variability in endline test scores than gender, age, years of experience in HIV care provision, and baseline score alone, and 3% of that variability could be attributed to the online CME courses.

**TABLE 7.** Percentage of Total Explained Variances Attributable to Intervention-Prompted Study Behaviors (n=48)

	Adjusted R^2^	% of Adjusted R^2^ Attributable to:		
		% of SMS Quizzes Correctly Answered	% of Daily Readings Accessed	Accessed Online CME Courses
Model 1. Gender, age, years of HIV care provision, baseline test scores	0.36			
Model 2. % of SMS quizzes correctly answered, gender, age, years of HIV care provision, baseline test scores	0.51	43		
Model 3. % of daily readings accessed, gender, age, years of HIV care provision, baseline test scores	0.40		10	
Model 4. Accessed online CME courses, gender, age, years of HIV care provision, baseline test scores	0.37			3

Abbreviations: CME, continuing medical education; SMS, short message service.

## DISCUSSION

The mCME intervention improved the knowledge of HIV clinicians in Vietnam by increasing the use of intervention-prompted study behaviors, namely, performance on the daily quizzes and accessing daily linked readings, but potentially also accessing the online CME courses. Unprompted changes in study behavior (i.e., searching for information outside of the embedded materials provided in the intervention itself) also may have played a role in improving clinicians' knowledge. In other words, the intervention was effective because it helped the participants to be better, more diligent, and more engaged students.

Starting from the high-level observation that the mCME version 2.0 intervention was effective at motivating study behaviors and led to gains on the endline exam compared with control participants, the analysis presented in this article furthers our insight into the likely mechanisms that mediated this result. Analysis of the intervention-prompted behaviors was facilitated by the click data tracked by software provided by the Vietnamese MOH. This allowed accurate measurement of accessing the quizzes, daily readings, and online CME courses. We found that performance on the daily quizzes most consistently predicted endline test performance, which strongly supports the strategy of using SMS quiz questions to motivate study behaviors. A key insight is that participation in the quizzes was less predictive than how well individuals performed on the quizzes. Because all our analyses controlled for baseline scores, we know that quiz performance was not a result of the participants' baseline knowledge. Because participation is a requirement to assess quiz performance, this suggests individuals who simply answered the SMS quiz question did not improve endline scores as much as individuals who used study resources to help answer the questions correctly.

Our findings support the strategy of using SMS quiz questions to motivate study behaviors.

Moreover, some of these factors interacted in synergistic ways. The daily readings and online CME courses mediated the strong performance on the daily quiz questions. When provided in conjunction with the SMS quizzes, students who accessed these 2 resources, particularly the daily readings, demonstrated improved performance on the quizzes and were the most successful on the endline exam relative to the rest of their cohort. This yields a conclusion that is in some sense very intuitive: the mCME version 2.0 intervention worked among those individuals who were cued by the daily quizzes into investing time into studying, and worked less well among students who only answered the SMS quiz question. As such, this largely supports the lateral learning model that guided this research project, which assumed that learning would largely result from investments of study time rather than from knowledge acquired directly from the daily quiz questions themselves. Since the control participants had the same opportunities to engage in self-study but rarely did so in practice, we can conclude that the daily prompts from the SMS quizzes were an effective way of motivating self-study, and that this strategy yielded meaningful results in terms of mastery of clinical knowledge.

Daily readings and online CME courses mediated the strong performance on the daily quiz questions.

Nonetheless, with all the intervention-prompted behaviors and demographic factors included in the analysis, only 43% of the total variance in endline score changes could be explained. Naturally, this leads us to study behaviors outside of those directly provided by the intervention—the independent study behaviors.

The independent study behaviors may have had a significant impact on endline scores. These data remain challenging to analyze because we could only ask about a limited number of self-study behaviors. Much of our analysis did not show statistically significant differences between the intervention and control groups, likely due to limitations in sample size. Additionally, these data were all taken from a single endline survey, which could not capture longitudinal changes in study strategies over the course of the intervention. Despite limitations in using surveys, data from the self-study behaviors we measured show distinct differences in study behaviors between the 2 groups. Resources that were associated with higher endline scores in the control group were often associated with lower endline scores in the intervention group and vice versa, suggesting that the intervention did not simply prompt higher usage of study resources but may actually have changed the way in which the participants approached the material or used their resources.

While it is impossible to know for certain why the intervention changed study habits, it is possible that exposure to many example questions along with easy access to good resources such as the online CME courses and daily hyperlinked readings provided insight into how the material was tested and allowed motivated students to better utilize reliable sources of information, such as scientific papers, over less reliable sources, such as consulting with colleagues or searching for information on Google. Some of these study behaviors actually appeared to be counterproductive, including general Google searches or consulting with co-workers for information.

The independent study behaviors still only explain a portion of the residual variance in endline scores. That should be no surprise: there are myriad ways in which individuals can study, and not all of these were captured by our survey. Nor are the semi-quantitative and subjective reporting of measured behaviors a perfect measure of study intensity. In future mCME studies, more thorough investigation into these independent study resources might better reflect changes in lateral learning that may have taken place as a result of the intervention. Future mCME studies should have a larger sample size for a more thorough assessment of lateral learning mechanisms.

## CONCLUSION

We conclude that mCME is a useful approach to improve clinicians' clinical knowledge and may be particularly useful in resource-limited settings where access to and/or support for in-person CME courses are limited. The mCME strategy is portable since the methodology can be applied to any content area, user group, or geography.^9^ Further, the mCME strategy is feasible; the groups supporting the mCME program will have to make available the software itself and the content that is to be broadcast, and participants will have to own a smartphone and have access to a mobile network.^9^ These technology-related requirements are seldom barriers in resource-limited settings since SMS messages nowadays use a fraction of the bandwidth required for media files and can function even where bandwidth is constrained.^9^ The success of the mCME version 2.0 intervention in Vietnam suggests that lateral learning, or the process of engaging in learning outside of the intervention but in response to the intervention, mediated the improvement in medical knowledge.^3^ Unpacking the “black box” has helped us to evaluate the underlying mechanisms by which mCME can improve medical knowledge.

## Notes

**Peer Reviewed**

**Funding**: Funding for the mCME project was provided by the Fogarty International Center at the U.S. National Institutes of Health (1R21-TW00911). Funding for mCME version 2.0 was provided as a supplement to the main award, with the stipulation that it focus on issues related to HIV/AIDS. The funders played no role in the conduct, analysis, or reporting of this study.
